# Cusp Fracture Resistance of Maxillary Premolars Restored with the Bonded Amalgam Technique Using Various Luting Agents

**DOI:** 10.1155/2009/946830

**Published:** 2008-12-25

**Authors:** Shivaughn M. Marchan, Larry Coldero, Daniel White, William A. J. Smith, Reisha N. Rafeek

**Affiliations:** ^1^School of Dentistry, Faculty of Medical Sciences, The University of the West Indies, St. Augustine, Trinidad, Trinidad and Tobago; ^2^Department of Physics, The University of Trinidad and Tobago, Couva, Trinidad, Trinidad and Tobago

## Abstract

*Objective*. This in vitro study uses measurements of fracture resistance to compare maxillary premolars restored with the bonded amalgam technique using a new resin luting cement, glass ionomer, and resin-modified glass ionomer as the bonding agents. *Materials*. Eighty-five sound maxillary premolars were selected and randomly assigned to one of five test groups of 17 teeth each. One group of intact teeth served as the control. The remaining groups were prepared to a standard cavity form relative to the dimensions of the overall tooth and restored with amalgam alone or a bonded amalgam using one of three luting agents: RelyX Arc (a new resin luting cement), RelyX luting (a resin-modified glass ionomer), or Ketac-Cem *μ* (a glass ionomer) as the bonding agents. Each tooth was then subjected to compressive testing until catastrophic failure occurred. The mean loads at failure of each group were statistically compared using ANOVA with a post hoc Bonferroni test. *Results*. It was found that regardless of the luting cement used for the amalgam bonding technique, there was little effect on the fracture resistance of teeth. *Conclusion*. Cusp fracture resistance of premolars prepared with conservative MOD cavity preparations is not improved by using an amalgam-bonding technique compared to similar cavities restored with amalgam alone.

## 1. Introduction

Amalgam has been shown to be an acceptable material with proven longevity for large posterior restorations [1]. The major disadvantage of amalgam, however, is its inability to bond to dental hard tissues preventing reinforcement or strengthening of prepared tooth structure. This lack of bonding necessitates the use of macromechanical retentive features which are inherently destructive and cause further weakening of the remaining tooth structure [2].

Resin bonding agents have been used for a number of years as an adhesive liner between dental amalgam and tooth structure. In vitro tests have shown that these adhesive systems form retentive bonds with amalgam that increase the shear bond strengths of amalgam to tooth and reduce microleakage [2, 3]. Other studies claim an increase in the fracture resistance of prepared teeth when restored with bonded amalgam [4, 5].

More recently, glass ionomer formulations have been tested as an adhesive liner with dental amalgam for the restoration of prepared teeth [6–8]. Certain glass ionomer formulations have demonstrated increased shear bond strength measurements of amalgam to dental hard tissue comparable to that achieved with resin systems [6, 8]. The fracture resistance of teeth restored with the bonded amalgam technique using restorative glass ionomer as an adhesive has been shown to be greater than that of prepared teeth alone and teeth restored with amalgam using a copalite liner [7].

The objective of this study was to assess the resistance of premolars with traditional MOD preparations to cuspal fracture when restored with amalgam that has been adhesively bonded to cavity preparations using various glass ionomer formulations as the adhesive liner compared to cavities restored with amalgam bonded with a resin luting liner and cavities restored with amalgam alone.

## 2. Methods and Materials

Three groups of teeth restored with the amalgam bonding technique, 1 group of intact teeth, and 1 group of teeth restored with amalgam only were included in this study. The luting cements used as amalgam bonding agents utilized in this study are shown in Table 1. A power calculation determined that a minimum of 10 teeth were needed in each group. A sample size of 17 per group was employed.

Eighty-five human maxillary premolars extracted for orthodontic reasons were collected and immediately stored in distilled water. The teeth were rinsed and any debris removed with a sonic scaler (Titan SW Scaler, Dental EZ Group, StarDental Division, Lancaster, Pa, USA). The teeth were embedded in individual cylinders of autopolymerizing polymethyl methacrylate (Fas-Tray, Harry J Bosworth Company, Stokie, Ill, USA) 1.0 mm short of the cementoenamel junction so as to mimic alveolar support for the tooth. After complete setting of the tray acrylic, the base was trimmed to expose a cross-section of the root in its apical one third. This allowed transmission of applied force entirely through tooth structure by preventing settling of the tooth within the tray acrylic during testing. The mounted teeth were randomly divided into five groups and stored in distilled water at 37°C.

### 2.1. Cavity Preparation

Mesio-occlusal-distal (MOD) cavities were prepared using a 245 bur (SS White, Lakewood, NJ, USA) in a high-speed handpiece (BELLAtorque, KaVo Dental Corporation, Lake Zurich, Ill, USA) with water spray. A new bur was used for every 5 teeth. One operator (*WS*) completed all the preparations. The dimension of each preparation was made proportional to the dimensions of the tooth to minimize variations as a result of the size of individual teeth.

The occlusal isthmus had a facial-lingual width of 1/3 intercuspal distance. The pulpal floor was 2/3 of the height of the cusps measured from the cementoenamel junction. The facial-lingual width of the approximal boxes was 1/3 the maximum facial-lingual width of the tooth. The gingival floor of the approximal box was established 1.0 mm above the cementoenamel junction. The depth of the approximal box (from gingival cavosurface to axial wall) was 1.0 mm. All internal line angles were rounded and no additional retentive features were cut.

Prior to actual cavity preparation, the outline of each cavity was drawn on the surface of the tooth as a preparation guide.

### 2.2. Adhesive Application and Restoration

#### 2.2.1. Control Group

The control group, of unprepared teeth, was returned to water for storage until testing.

#### 2.2.2. Amalgam Group

Number 1 Tofflemire band (Sullivan-Schein Dental, Melville, NY, USA) was placed in a universal Tofflemire matrix retainer (Teledyne Getz, Elk Groove Village, Ill, 60007) and secured around the tooth. The cavity walls were dried using clean compressed air. High copper, platinum modified, precapsulated, spherical amalgam (Logic, SDI Ltd, Australia) was mechanically triturated (Wig-L-Bug, Crescent Dental Mfg Co, Lyons, Ill, USA) following the manufacturer's instructions followed by hand condensation into the prepared cavity. The matrix band was removed and the amalgam was carved and finished following the normal anatomical contours of the tooth.

#### 2.2.3. Adhesive Application

The cavity walls of the remaining three groups were coated with one of three luting cements: RelyX Arc (3M ESPE, St. Paul, Minn, USA), RelyX luting (3M ESPE, St. Paul, Minn, USA), or Ketac-Cem *μ* (3M ESPE, St. Paul, Minn, USA). All materials were dispensed and handled according to the manufacturer's instructions.

Following cement application, each tooth was immediately restored in the same manner as the amalgam group, with the amalgam being condensed into the cavity while the adhesive liners were still unset. The matrix band was removed to allow carving of the amalgam to normal anatomical contours and removal of any adhesive flash at cavosurface margins. The exact handling techniques utilized for each adhesive material were as follows.

**RelyX Arc**

The cavity was etched with 37% phosphoric acid for 20 seconds using the total etch technique. The acid was then thoroughly rinsed for 20 seconds and the cavity was dried, but not desiccated, with compressed air. Single bond (3M ESPE, St. Paul, Minn, USA) was applied to the cavity using a microapplicator tip and dried for 2 seconds with compressed air, leaving glossy cavity surfaces. The dentine bonding agent was then light cured for 10 seconds using visible blue light (Optilux, Demetron/Kerr 21 Commerce Drive, Danbury, Conn, USA) at 460 *η*m wavelength. The RelyX Arc resin luting cement was dispensed in equal amounts and mixed for 10 seconds and applied to the cavity using a microapplicator brush. Amalgam was condensed and finished as previously described.

**RelyX Luting**

The cavity was washed with distilled water and dried with compressed air from the air-water tip, preventing desiccation of tooth tissue. The powder was fluffed and the standard powder: liquid ratio of 1.6:1, by weight, was dispensed on a glass slab. Mixing was completed with the aid of a metal spatula for 30 seconds. The mixed cement was applied to the cavity walls using a microapplicator brush. Amalgam was condensed and finished as previously described.

**Ketac-Cem *μ***

the cavity was washed with distilled water and dried with compressed air from the air-water tip, preventing desiccation of tooth tissue. The powder was fluffed and the standard powder: liquid ratio of 3.8:1, by weight, was dispensed on a glass slab. Mixing was completed with the aid of a metal spatula for 30 seconds. The mixed cement was applied to the cavity walls using a microapplicator brush. Amalgam was condensed and finished as previously described.

### 2.3. Testing

**Figure 1:**
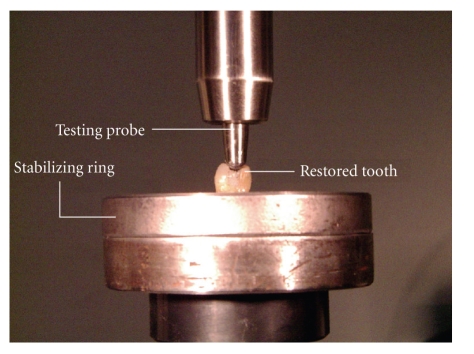
Photograph of restored tooth within compressive testing probe in contact with the cuspal inclines.

All specimens (control and restored teeth) were stored in 100% humidity at 37°C for 14 days prior to testing. The teeth were placed in a stabilizing ring, to avoid movement, and tested in compression in a Hounsfield H50KS tensometer (Tinius Olsen Ltd, Redhill, Surrey, UK) using a rounded, stainless steel, testing probe 5 mm in cross-section. The profile of the testing probe ensured contact with buccal and lingual inclines of cusp triangular ridges and not the amalgam restoration (see Figure 1). The teeth were preloaded to a maximum force of 250 N at a speed of 5 mm/min for 10 times (with the speed of return at 50 mm/min), prior to catastrophic testing. The preloading was performed to simulate clinical situations where the occlusal surface of the tooth is subjected to repeated chewing forces. Following this preloading procedure, the teeth were tested to failure at a speed of 0.5 mm/min until cusp fracture occurred.

Means, with standard deviations, were calculated for each group. Group means were analyzed using a one-way analysis of variance (ANOVA) followed by a post hoc Bonferroni multiple comparison test.

## 3. Results

During pretesting, a number of specimens were lost in each group due to early fracture. Two teeth were lost in the sound group, 1 in the amalgam group, 1 in the RelyX Arc group, none in the RelyX luting group, and 2 in the Ketac-Cem *μ* group. The analysis of variance revealed that there were no significant differences in fracture resistance of teeth restored with amalgam alone or amalgam bonded with various luting cements (*P* > .05). There was however a highly significant difference between the intact teeth (control group) and all the restored groups (*P* < .05). The means and standard deviations, together with statistical significance, for the remaining teeth are presented in Table 1. This study did not attempt to characterize the fracture patterns of the failed specimens.

## 4. Discussion

Various studies have shown that prepared teeth fracture more readily than sound intact teeth [9, 10]. Ideally any material that is used to restore missing tooth structure should reinforce the tooth and minimize risk of cuspal fracture.

In this study, the difference in resistance to catastrophic fracture between the sound (unprepared) teeth and restored teeth was highly significant. This supports previous findings that demonstrate the deleterious effect that cavity preparation has on the fracture resistance of posterior teeth [11].

The results of this study showed no statistically significant differences in cusp fracture resistance between teeth restored with the bonded amalgam technique that utilized either a resin-based adhesive (RelyX Arc), glass-ionomer adhesive (Ketac-Cem *μ*), or resin-modified-glass ionomer (RelyX luting) and a conventional technique using amalgam alone. These findings are contrary to those of other investigators, who have shown an increase in fracture resistance of teeth restored with the bonded amalgam technique using both resin and glass-ionomer luting cements when compared to teeth restored with amalgam alone [7, 12]. This difference may be explained by the size of the cavity preparation utilized in this study. The isthmus preparation dimensions of 1/3 the intercuspal width and the facial-lingual width of the approximal boxes at 1/3 the maximum facial-lingual width of the tooth could be considered conservative. This resulted in a significant portion of coronal dentin remaining, the mechanical properties of which contributed to similar fracture resistance values within the tested groups. Indeed, researchers that use more conservative cavity preparations have found that fracture resistance is not improved regardless of the restorative material or technique used [13]. In a similar study where amalgam was bonded into cavities of similar dimensions using filled and unfilled resin systems, it was noted that there was no increase in the fracture resistance of restored teeth [13]. This may explain the unexpected results shown by the sole resin system used, RelyX Arc, to bond amalgam in this particular study.

Investigators who use larger cavity preparations have consistently shown that the use of the amalgam-bonding technique significantly contributes to the fracture resistance of the teeth [5, 14].

This particular research produced large variations in the standard deviation values among the tested groups. This may be attributed to the normal mechanical and anatomical variations of natural teeth, including, the ratio of enamel to dentin, cusp position and angulation, cusp height, and undetectable flaws. Another explanation of the high standard deviations may be due to the catastrophic forces applied in vitro until failure occurred. Such in vitro forces are applied at a constant direction and speed until failure occurs, which rarely mimics failure intraorally.

Although the glass ionomer and resin-modified glass-ionomer luting cements used as adhesives for amalgam alloy did not increase the fracture resistance of the prepared teeth in this particular study, it must be noted that both conventional glass ionomer and resin-modified materials have shown increased measurements of shear bond strengths at the dentin/amalgam interface which has the effect of increasing retention of amalgam to tooth structure, thus diminishing the reliance on macromechanical features which has the overall effect of conserving tooth tissue [6–8].

## 5. Conclusion

Within the limitations of this study, the conclusion may be drawn that maxillary premolars restored with the bonded amalgam technique using glass ionomer, resin-modified glass ionomer, and resin luting cements do not improve measurements of fracture resistance. The clinical relevance of this is that amalgam bonded into conservative MOD preparations of premolars does not offer increased strength to the tooth and restoration but only unnecessarily increases the complexity of the clinical procedure.

## Figures and Tables

**Table 1:** Mean fracture strengths and standard deviations of tested teeth.

Group	N	Mean (S.D.)	ANOVA/post hoc Bonferroni comparison
Control (intact)	15	2070.9(317.02)	A
Amalgam	16	917.4(198.04)	B
RelyX Arc	16	834.5(323.73)	B
RelyX Luting	17	869.6(299.39)	B
Ketac-Cem *μ*	15	917.8(344.73)	B
Similar letters are not significant at *P* = .05			
